# Predictability awareness rather than mere predictability enhances the perceptual benefits for targets in auditory rhythms over targets following temporal cues

**DOI:** 10.1371/journal.pone.0284755

**Published:** 2023-10-27

**Authors:** Miriam Heynckes, Kim Hoffmann, Elia Formisano, Federico De Martino, Peter De Weerd

**Affiliations:** 1 Department of Cognitive Neuroscience, Faculty of Psychology and Neuroscience, Maastricht University, Maastricht, The Netherlands; 2 Maastricht Centre for Systems Biology, Maastricht University, Maastricht, The Netherlands; University of Western Ontario, CANADA

## Abstract

Sounds following a cue or embedded in a periodic rhythm are processed more effectively than sounds that are part of an aperiodic rhythm. One might also expect that a sound embedded in a periodic rhythm is processed more effectively than a sound following a single temporal cue. Such a finding would follow the theory that the entrainment of neural rhythmic activity by periodic stimuli renders the prediction of upcoming stimuli more efficient. We conducted a psychophysical experiment in which we tested the behavioral elements of this idea. Targets in periodic and aperiodic rhythms, if they occurred, always appeared at the same moment in time, and thus were fully predictable. In a first condition, participants remained unaware of this. In a second condition, an explicit instruction on the temporal location of the targets embedded in rhythms was provided. We assessed sensitivity and reaction times to the target stimuli in a difficult temporal detection task, and contrasted performance in this task to that obtained for targets temporally cued by a single preceding cue. Irrespective of explicit information about target predictability, target detection performance was always better in the periodic and temporal cue conditions, compared to the aperiodic condition. However, we found that the mere predictability of an acoustic target within a periodic rhythm did not allow participants to detect the target any better than in a condition where the target’s timing was predicted by a single temporal cue. Only when participants were made aware of the specific moment in the periodic rhythm where the target could occur, did sensitivity increase. This finding suggests that a periodic rhythm is not automatically sufficient to provide perceptual benefits compared to a condition predictable yet not rhythmic condition (a cue). In some conditions, as shown here, these benefits may only occur in interaction with other factors such as explicit instruction and directed attention.

## Introduction

Acoustic stimuli often have temporal structure that can be leveraged by the brain. Temporal structure may be rhythmic as in the sounds produced by a metronome (termed isochronous), or (quasi-) rhythmic as in speech or music. Temporal structure can also take the form of a temporal association between a cue and a stimulus presented after a given time interval, as in thunder following lightning. It is generally accepted that the efficiency with which stimuli are processed benefits from them being embedded in temporal structures. The present paper investigates to which extent instructions that inform participants on specific features of the temporal structure, which represent a manipulation of top-down attention, contribute to these processing benefits. In the context of this manipulation, benefits for auditory processing will be compared between target stimuli temporally associated with a single cue, and targets embedded in periodic or aperiodic rhythms.

Temporal associations consist of a warning cue followed by a potential target and have typically been investigated using temporal foreperiod tasks [1, 2] and temporal cueing tasks [3, 4]. In constant foreperiod tasks, a warning signal and target stimulus are presented, between which the time interval remains constant within a block of trials and is varied between blocks. These tasks have shown that temporal prediction efficiency is optimal at shorter time intervals and diminishes with longer intervals between warning signal and cue, as evidenced by increased reaction times [2] and lower accuracy [5]. In temporal interval cueing tasks that use two cueing intervals, a valid temporally predictive cue (compared to the invalid cue) considerably improves reaction times and accuracy for targets at the shorter of two possible intervals [6, 7, for a review see 4]. If two cueing intervals are used, participants are explicitly instructed on the cue-target relationships, and it can therefore be assumed that participants in these studies are aware of the time interval value following each cue, predicting the occurrence of the target stimulus. Similarly, we assume that if one constant cue interval is used within a block, participants are aware of its explicit timing for execution of the task. This may lead to top–down attention focused to a specific moment in time, thereby enhancing processing of stimuli presented around the expected time. The temporal predictability of targets afforded by a temporal cue preceding a target may also be implicit, which occurs when the temporal interval between cue and target on any given trial is drawn from a distribution. Under these conditions, it has been shown that the less predictable the cue-target relation becomes (i.e. the wider the distribution), the more listeners’ reaction times are slowed [8, 9].

Rhythmicity is another form of temporal structure that can be leveraged by the brain. The properties of stimuli are processed more efficiently by virtue of them being preceded by a periodic rhythm. In line with this idea, seminal work by Jones and colleagues [10–12] has shown accuracy benefits for targets occurring in-phase compared to out-of-phase with a preceding rhythm. Perceptual benefits of periodic rhythms can be attributed to the entrainment of ongoing neural oscillations [13–15], where the high-excitability phase of neural populations become aligned to the timing of relevant events. Although the present study reports only behavioral data, entrainment theory provides a useful framework within which to consider benefits of periodic over aperiodic rhythms. When entrainment is present, stimuli presented periodically should entail perceptual benefits for targets occurring in phase with the stimulation, even after the stimulation has ended, which has been demonstrated by a large number of studies [10, 16–22]. A processing advantage of periodicity has been shown even when the periodic structure was task irrelevant [23] or when the task required focusing on a different (predictive) stimulus feature (e.g. stimulus color) [18, 24]. These suggested automatic benefits of rhythms have proven advantageous in speeded response tasks for frontal lobe patients in whom temporal associative processing of a cue and a stimulus was impaired [21]. While these findings suggest that rhythmic structure is processed in a primarily bottom-up stimulus driven fashion, an alternative explanation for the perceptual benefits of periodicity is that it guides top-down attention [11]. According to this hypothesis, (rhythmic) attention permits processing targets that are in phase with the stimulation more efficiently, explaining the advantage of periodic versus aperiodic stimulation. Thus, whether auditory neural activity automatically entrains to rhythmic input or whether its entrainment is under active top-down control remains a topic of debate [25, 26]. Previous studies comparing a rhythm to a temporal cue have shown that the expectancy profile to a preceding rhythm is sharper than that produced by a single cue (Experiment 4, [10]) and that target perception benefits more from preceding rhythms than from preceding single cues [20]. Combining a rhythm and a temporal cue has been shown to have an additive effect in reducing reaction times [22], suggesting perhaps a similarity in (part of) the underlying mechanisms [27].

Here, we aim to gain insight into factors influencing the benefits induced by periodicity, by manipulating awareness (through task instruction) of the position in the rhythm where targets may be presented. We opted for having a constant location within a rhythm for potential target presentation, and we ran one study in which participants were explicitly informed about the location in the sequence where the target might occur, and another study in which they were not informed. To the best of our knowledge, the detection of targets embedded at a fixed and thus predictable position of which listeners are however unaware, has not yet been compared to the detection of the same targets in listeners benefiting from the awareness of target predictability due to task instruction. We compared the perceptual benefits of periodic rhythms to benefits of aperiodic rhythms and those of temporal interval cueing. The effect of an instruction was tested in both the periodic and aperiodic stimulus conditions, but not in the temporal cueing conditions (see Materials and methods). Using this experimental design, we aimed to answer two principal questions. (1) Does a periodic rhythm lead to improved behavioral performance relative to a temporal cue and an aperiodic rhythm? (2) Does instructing in which position of the periodic and aperiodic rhythm to expect the possible presentation of a target affect the behavioral benefit of a rhythm compared to a temporal cueing interval? Our main finding was that the expected superiority of periodic rhythms over single cueing only emerged when participants were explicitly informed about the constant location of the targets within the periodic stimulus sequence.

## Materials & methods

### Experimental design

The main task of the participants was to detect a target in a quintet of rapidly presented sounds (See *Stimuli* for details). The experimental design consisted of a single within-subjects variable ‘temporal structure of stimuli’ and a single between-subjects variable ‘instruction’. For the temporal structure of the stimuli, there were three conditions in which the presentation of a target within a quintet occurred in (1) a periodic sequence of multiple quintets, (2) an aperiodic sequence of multiple quintets, or (3) following a single cued quintet. The between-subjects variable ‘instruction’ had the purpose of manipulating awareness of the constant location of the target in the periodic and aperiodic stimulus sequences, by applying an instruction in one group explicitly telling participants when to expect the target, and not having that instruction in the other group of participants (see Instruction for details). The difference in instruction was applied to both the periodic and aperiodic conditions, but not to the single cue condition, because in the latter condition there was no uncertainty as to which quintet in the stimulus could contain the target. Nevertheless, whenever we refer to the experimental design, we will maintain the terminology of an instruction variable with instructed and no-instruction condition, and of a temporal stimulus structure variable, with periodic, aperiodic, and single-cue conditions.

All stimulus presentation scripts were written in Matlab (The MATHWORKS Inc., Natick, MA, 234 USA), using the Psychophysics toolbox [28] and are publicly available at [29]. Participants sat in front of a computer placed within a sound-attenuated chamber while listening to the stimuli delivered through Sennheiser HD650 headphones. Instructions to fixate on the cross and to respond as fast and accurately as possible upon hearing the target were presented on the computer screen. Responses were collected with a standard keyboard. Participants were asked to detect a target; a temporal shift of a narrow-band sound (see *Stimuli* for details). During a trial, and up to 1s after a sound finished, participants could indicate whether they perceived a target. After a variable interval the next trial was initiated. Participants performed a total of 432 trials, divided over 6 blocks. Each block contained three mini-blocks in which the temporal structure was constant, with a mini-block consisting of 24 trials of periodic stimuli, followed by a mini-block of 24 trials aperiodic stimuli and a mini-block of 24 cue stimuli. The order of mini-blocks within each block was fully counterbalanced within each participant and randomized across participants. The temporal shift target occurred in 75% of the trials (see details on temporal shift target in the stimuli section, subsection ‘Targets in the quintets’).

#### Instruction

We used the following verbal instruction to manipulate awareness of the participants of the fact that the target was predictable: ‘Targets are much more likely to occur on the 10th position. Using counting may be beneficial in performing the task’. The instruction applied to the periodic and aperiodic conditions only, and was effective in the group where it was applied as confirmed by a post-experiment questionnaire. After 2 blocks (out of 6 blocks in total the experimenter checked in with the question, whether counting helped the participant? This question served as a reminder to the participant. The verbal response of the participant was not recorded. However, in the post-experiment questionnaire all participants reported using counting as a strategy to perform the detection task. That the instruction was effective as an experimental manipulation highlights that detecting a temporal shift was a difficult perceptual task. As a consequence, in the uninstructed condition most participants remained unaware (as assessed by the post-experiment questionnaire) of the constant position of the target within periodic and aperiodic stimuli. A subset of the participants in the no-instruction condition showed some minimal awareness of the fact that the target was not fully randomly positioned in the stimulus, but none of them reported awareness of the exact location of the target in the stimulus. Specifically, 8 participants reported noticing targets occurring more frequently towards the end of a stimulus, while 2 participants reported noticing that the target was always at the same position, yet were unable to count and name the specific quintet position of the target. This contrasts with the instructed conditions, in which participants received a verbal instruction when (at which position) to expect a target within both the periodic and aperiodic rhythm.

### Participants

In the no-instruction condition, we collected data from 24 participants of which 20 participants (8 male, 12 female) were included in the final analysis. Two participants were excluded due to failure to comprehend and/or execute the task and two participants due to an abnormal audiogram (>20 dB at audiometric test frequencies) assessed prior to testing. In the instructed condition, we collected data from 17 participants, 2 were excluded due to failure to comprehend and/or execute the task, resulting in a total of 15 participants (1 male, 14 female). Participants were screened for normal hearing (ranging between 0 and 15 dB) at audiometric test frequencies from 0.25–8 kHz. The sample size of each condition and size of the general detection effect across conditions were comparable to sample size and effect size in our previous study (Fig 4A) [30]. The Ethics Review Committee of the Faculty of Psychology and Neuroscience (ERCPN) at Maastricht University granted approval for all studies and all participants gave written informed consent.

### Stimuli

Acoustic stimuli were created in Matlab (The MATHWORKS Inc., Natick, MA, 234 USA) at a 44.1kHz sampling rate, 16-bit resolution.

#### Macro-temporal structure

**Fig 1:**
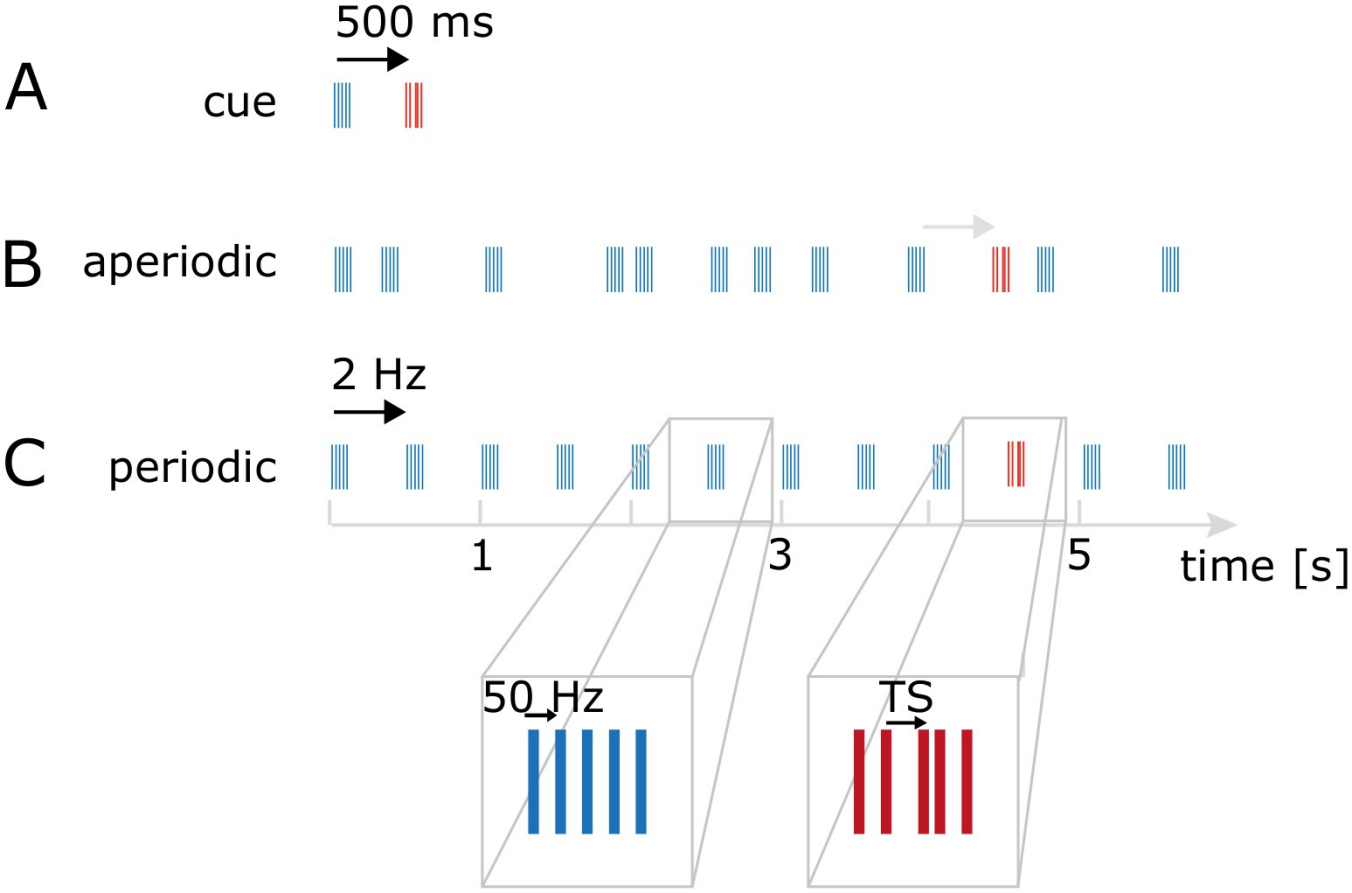
Temporal structure of stimuli. **A**. a temporal cue precedes the target (TS). **B**. Aperiodic rhythm with TS. Quintet TS-1 precedes at 2 Hz ISI. **C**. Periodic rhythm at 2 Hz with TS. Five sounds repeating at 50 Hz (10ms ISI) create a quintet (*inset 1*). Target stimuli (*inset 2*) had a different temporal structure: the third sound in a quintet was temporally shifted between 1.5–7 ms, depending on a subject’s perceptual threshold.

Three types of acoustic stimuli with different temporal structure were created: periodic and aperiodic rhythms of 5.5 s length, and a temporal cue condition of 700 ms length (see Fig 1). All stimuli had an (average) interstimulus interval (ISI) of 500ms, corresponding to a 2 Hz rate at which sound quintets were repeated. To create the aperiodic rhythm with a total stimulus duration of 5.5 seconds, on each trial we randomly sampled ISI (in ms) from a vector (x = [110 130 210 230 280 445 740 700 965 1190], with a mean of 500 ms).

#### Quintets (Micro-temporal structure)

The quintets consisted of five narrowband sounds of 10ms duration that were presented at a frequency of 50 Hz (i.e. an ISI of 10 ms). The narrow passband was centered on a carrier frequency of 1100 Hz (ERBS = 4; [31]), consisting of a summation of 21 sinusoids, each with a random phase onset.

#### Targets in the quintets

In a subset of quintets, the 3rd of five narrowband sounds was shifted in time. Participants had to report the presence of the temporal shift whenever it occurred in a quintet. Per participant the size of this shift was determined by a staircase procedure, converging on a 70% detection threshold (2 down 1 up) and ranged between 1.5 and 7ms in ten logarithmically spaced steps [32]. The termination criterion was after 200 trials or 15 reversals and the perceptual threshold was computed as the mean over the last 12 reversals. S1 Fig shows the staircase performance over the course of the experiments across participants.

The TS distribution over the quintets was non-uniform. In the temporal cueing condition, a temporal shift, if present, occurred at the second (cued) quintet. The temporal shift in the periodic and aperiodic conditions (if present) occurred at the 10th quintet (i.e. after ~4.5 s). Additionally, in the aperiodic condition the interval between the target quintet and the preceding quintet was kept constant at 500ms.

### Statistical analysis

All analyses were conducted in MATLAB 2017a (The MATHWORKS Inc., Natick, MA, 234 USA). The data and analysis code can be found at [33], we have applied the analysis previously in [30]). Statistical group analyses were carried out using generalized linear mixed models (GLMM), which were assessed using likelihood ratio tests. The independent variables were temporal structure (3 within-subject levels: periodic, aperiodic, cue) and instruction condition (2 between-subject levels of either receiving or not receiving an instruction when (at which position) to expect a target within the periodic and aperiodic rhythm. For each participant, we computed sensitivity (d prime) as well as mean log- reaction time (logRT) for correct trials, forming our dependent variables. Reaction time was calculated relative to temporal shift target onset. Traditionally, d’ is estimated by counting the frequency of an observer reporting ‘yes’, conditional on the presence and absence of the signal (i.e. the hit and false alarm rates) and taking the difference of these values on a z-transformed scale. In the present work, d’ scores and model parameters were estimated in one step, by linearly modeling the behavioral outcomes of the detection task (yes & no) with a predictor target coding for the presence or absence of the target and employing an inverse Gaussian link (probit) function, rather than estimating d’ on each condition separately and feeding the estimated values to a second level analysis. Within this framework, d’ can be estimated by linearly modeling the behavioral outcomes (i.e. ‘yes’ or ‘no’) with a predictor *X* coding for the presence or absence of the target (see right side of Eq (1); and “Target” predictor in S1 Table). To fit an equal-variance Gaussian signal detection model an inverse Gaussian (probit) link function is used, where *g* is the link function and *X* represents the presence or absence of the signal.


$$g\left(E\left[Pr\;Pr\;\left(Resp{=}^{\prime }Yes^{\prime }\right)\right]\right)=\beta_{0}+\beta_{1}X$$ (1)

**Fig 2:**
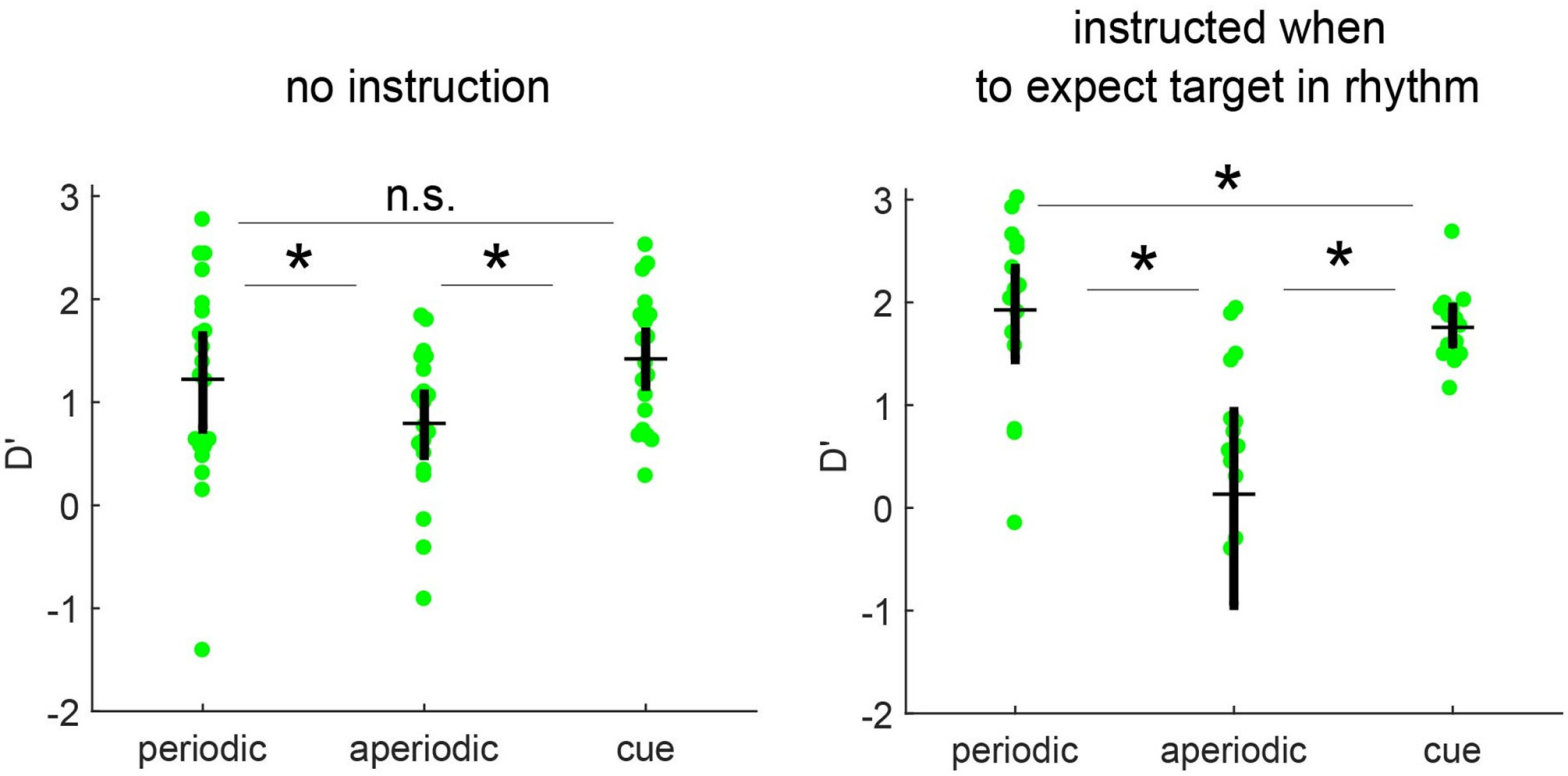
Instruction when to expect a target improves sensitivity in periodic rhythm compared to a temporal cue. **A**. In the no-instruction condition, participants received no instruction on possible target positions within the temporal sequences and remained unaware that targets occurred always at the same position. In this case sensitivity did not differ between a periodic rhythm and a temporal cue. **B**. When participants were instructed on the temporal contingency of target occurrence in periodic and aperiodic stimuli (participants were instructed in which position to expect the possible presentation of a target), a higher sensitivity for the periodic rhythm compared to temporal cue (as well as aperiodic) occurs. Filled circles depict d’ per participant. Black horizontal line shows the group mean. Errorbars depict bootstrapped confidence intervals (at subject-level). Note: An outlier is not visible. To see all single subject data points, S2 Fig displays the same data on a different axis, using a discontinued y-axis for visualization purposes.

When the signal is absent (i.e. *X* = 0), *β*_0_ provides an estimate of the normal quantile of the false alarm rate. When the signal is present (i.e. *X* = 1), *β*_1_ reflects the difference between hit and false alarm rate on the probit scale (hence, the difference between z-scaled hit and false alarm rates), or d’. The different experimental conditions were then added as predictors, and the estimated d’ for each of these conditions (hence our effect of interest) is described by the interaction term between *X* (‘target’) and the respective condition predictor (see [34], chapter 3.3.5). Unstandardized effect sizes (betas) are reported in units of the dependent variable (d‘or logRT), allowing for a meaningful comparison, in line with general recommendations on how to report effect sizes in psychological research [35]. This statistical framework (GLMM) extends multiple linear regression to non-normal data such as count data and binary outcomes and is more suited to handle extreme cases (100% hits or 0% false alarms). The full notation of the models is found in the S1 Table. Post hoc tests were performed by specifying contrasts and corrected for multiple comparisons (where applicable) using Bonferroni correction. For our visualization, we estimated d’ as it is traditionally computed. Standard errors of d’ group effects displayed in Fig 2 were obtained by non-parametric bootstrap sampling of estimated d’ values, carried out at the subject level (N = 1000). The mean was used as a measure of central tendency around which 95% confidence intervals were created.

## Results

### Instruction improves sensitivity of auditory target perception in periodic rhythm compared to a temporal cue

We investigated the benefits in perceptual sensitivity and reaction times associated with different temporal structures of sounds (periodic, aperiodic and cue) as a function of the (between-subjects) instruction condition. The instruction condition informed participants that counting (in periodic and aperiodic stimuli) may help them to detect targets as they were more likely to occur in a specific position in the rhythmic sequence, while participants in the no-instruction condition did not receive such an instruction. We fitted a GLMM to the sensitivity data for target detection. This analysis showed a significant three-way interaction of target, instruction and temporal structure on d’ (F (2,198) = 48,55, p < 0.001), showing that the effect of temporal structure on sensitivity differed between the two instruction conditions. Follow-up tests showed that all pairwise differences among the three temporal structures significantly differed between the instruction conditions. First, the difference between periodic and aperiodic rhythm (given the target predictor) differed significantly between the instruction and no-instruction conditions (t (1, 198) = 49.84, p< 0.001). Second, the difference between the temporal cue and aperiodic rhythm (given the target predictor) differed significantly between the instruction conditions (t (1, 198) = 38.43, p< 0.0001). Third, there was also a significant difference between the temporal cue and periodic rhythm conditions (given the target predictor) (t (1, 198) = 7.01, p< 0.05) between the instruction conditions. Because of the significant three-way interaction of target, instruction and temporal structure, we analyzed the interaction of target and temporal structure separately for the two instruction conditions. In both the instructed and the no-instruction condition, d’ varied significantly as a function of the temporal structure (Fig 2A, *no Instruction*; F (2, 114) = 25.469, p< 0.001; Fig 2B, *with Instruction*; F (2, 84) = 170.66, p< 0.001). Follow-up comparisons of the temporal conditions in the condition without instruction showed a d’ benefit for periodic rhythms over aperiodic rhythms (Fig 2A, beta = -0.3663; t (1, 114) = 20.98, p< 0.001). Similarly, a d’ benefit was present of periodic over aperiodic rhythms in the condition with instruction (Fig 2B, beta = -1.55; t (1, 84) = 241.14, p< 0.001). A d’ benefit was also observed for the temporal cue over the aperiodic rhythm both without instruction (Fig 2A, *beta* = -0.5646; t (1, 114) = 48.87, p< 0.001) and with instruction (Fig 2B, *beta* = -1.42; t (1, 84) = 228.05, p< 0.001). These findings replicate our previously reported results [30].

Crucially, whether or not participants received an instruction on the position of target occurrence within periodic rhythms compared to a temporal cue had a significant effect on their detectability. When the temporal location of potential target presentations within a periodic rhythm was not made explicit by instruction, sensitivity due to the periodic rhythm did not differ compared to that afforded by a single temporal cue (Fig 2A, *no Instruction*; beta = 0.217; t (1, 114) = 1.37; p>0.5). In contrast, when target predictability was made explicit by instructing participants, sensitivity in the periodic condition was significantly higher compared to a temporal cue (Fig 2B, *with Instruction*; beta = 2.7464; t (1, 84) = 173.78; p<0.001).

### Limited effect of instruction on processing speed of targets in periodic and aperiodic rhythms

**Fig 3:**
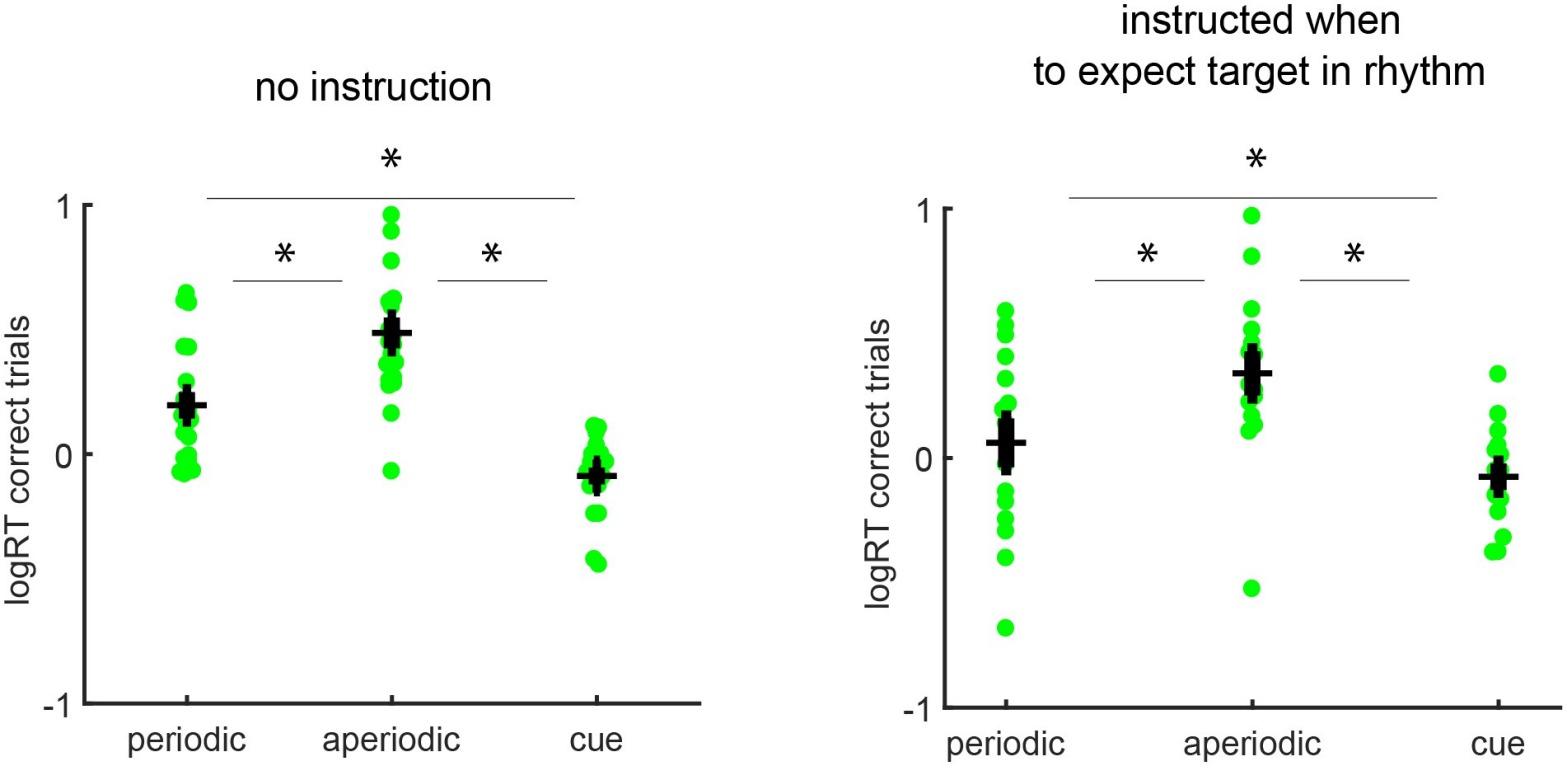
Log reaction times are fastest following the temporal cue, independent of instruction. (**A**) Participants had received no instruction on target position, but an implicit temporal prediction of the target position within the temporal rhythms and remained unaware that targets occurred at the same position. (**B**) In the instructed condition participants were instructed in which position to expect the possible presentation of a target in the periodic and aperiodic rhythm. Whether or not an instruction was provided did not significantly interact with effects of temporal structure on logRT. Main effect of temporal structure was therefore analyzed across the two instruction conditions. A temporal cue led to the fastest logRTs, followed by the periodic and aperiodic conditions. Filled circles show single participant mean logRTs on correct trials. Black horizontal line shows the group mean. Error bars depict SEM.

We fitted a GLMM to the response times obtained per temporal condition and instruction conditions. The analysis of reaction times showed a non-significant interaction of instruction and temporal condition (F (2,99) = 0.99; p>0.05). Providing an instruction did not have a significant effect on reaction times, compared to when no instruction was provided (Fig 3A and 3B, F (1,99) = 2.72; p = 0.1). The temporal condition had a significant effect on reaction times in both instruction conditions (Fig 3A and 3B, F (2,99) = 24.2; p<0.001). Follow-up tests showed that participants responded faster to targets following a single temporal cue, than to targets in periodic rhythms, (Fig 3A and 3B, F (1, 99) = 15.44, p<0.001) as well as compared to aperiodic rhythms, (Fig 3A and 3B, F (1, 99) = 81.56, p<0.001). Moreover, comparisons revealed faster correct responses for periodic than aperiodic rhythms (Fig 3A and 3B, F (1, 99) = 43.83, p<0.001).

## Discussion

Temporal expectations facilitate sensory processing and perception, and they play an important role in perceiving the outside world [4]. Currently, little is known about whether shared or separate mechanisms contribute to temporal expectations based on different temporal structures of sounds. Specifically, it is unclear if periodic rhythms and temporal interval cues affect behavioral performance in similar ways, relative to aperiodic rhythms. Moreover, to what extent these effects rely on task instruction and attention is also still unclear.

In the present study, we investigated the effect of an instruction informing participants about the predictability of a target on reaction times and sensitivity (d’) in a difficult temporal detection task. The effect of the instruction was tested in periodic and aperiodic stimulus conditions (but not in a single cue condition where the instruction was never given, see Materials and methods). Specifically, in the instruction conditions, we either gave no instruction about when to expect a possible target in any of the three temporal conditions (no instruction) or provided an instruction on when to expect a target within a periodic or an aperiodic rhythm (instructed condition). In both instruction conditions and in all temporal conditions, targets (if they occurred) were always presented at the same temporal position within periodic and aperiodic rhythms (i.e., the 10^th^ quintet, see Fig 1), or after a fixed time interval if following a single temporal cue. With this design, we set out to investigate (1) whether a periodic rhythm led to improved sensitivity relative to a temporal cue and an aperiodic rhythm and (2) whether instructing in which position of the periodic and aperiodic rhythm to expect a possible target presentation affected the behavioral benefit of a periodic rhythm compared to a temporal cueing interval and an aperiodic rhythm.

With respect to perceptual sensitivity, we showed in both instruction conditions that periodic rhythms led to improved sensitivity compared to aperiodic rhythms, and that the size of this effect was increased when instructions informed participants on specific features of the temporal structure (representing a manipulation of top-down attention) were provided. This finding is in line with the frequently reported behavioral benefit of periodic stimulation and indicates that this benefit can be magnified with an instruction. Interestingly, we also found in both instruction conditions that single cues led to improved sensitivity compared to aperiodic rhythms, and that the size of this effect was also increased in the instruction condition. This is interesting as in the cue condition no instruction is ever given. Since the three stimulus conditions were presented in short blocks of trials with blocks randomized in order within participants, participants presented with instructions in periodic and aperiodic conditions may have become generally more alert so that the instruction benefit generalized also to the no-instruction single temporal cue condition.

Importantly, in the no-instruction condition, when comparing the periodic rhythm and temporal cue, we showed that both a rhythm and a temporal cue led to a similar sensitivity when participants were not receiving any explicit instruction of when to expect a target, replicating our previous findings [28]. However, in the instruction condition, we found improved behavioral detection performance in the periodic rhythm compared to the temporal cue. This shows that the target detection benefit in periodic stimuli relative to single-cue stimuli is crucially dependent on the instruction.

The entrainment of ongoing oscillations can be seen as a theoretical framework for the mechanisms underlying the bottom-up benefits of rhythmicity [25]. Here, we show that attention can boost expectations elicited by periodic stimulation leading to enhanced sensitivity in target detection. In the absence of an instruction, the periodic rhythm provided no behavioral advantage in sensitivity compared to a temporal cue. This argues against the idea that there are always automatic benefits of rhythmic stimulus presentations. Therefore, our results may suggest that, in the absence of instruction, entrainment of oscillations alone would not provide a vehicle for the generation of predictions that are more efficient than the predictions provided by a single cue of the temporal interval after which a target follows. We suggest that explicit instruction allowed participants to intentionally use the rhythm, guiding attention to a specific moment in time [36]. The focusing of attention to a precise point in time in the periodic sequence may have been aided by the accumulated evidence of the precise length of inter-stimulus intervals, providing an advantage over both aperiodic and interval cue conditions. Thus, the temporal narrowing of the window of attention may have facilitated behavioral sensitivity in addition to the benefit of in-phase tone presentation in the periodic condition. This suggestion is corroborated by previous findings showing behavioral beneficial effects on reaction times to targets through the intentional use of a rhythm [18], and by the previously reported link between attention and changes in sensory gain associated with entrainment [14, 37]. In addition, proposals that temporal predictions (instantiated through a rhythm or a temporal cue) modulate ongoing oscillations through a top-down reset [27] are compatible with this view. In the same vein, by inserting cues within ongoing rhythms, advantages of attentional allocation for the detection of targets embedded in rhythms have also been demonstrated, crucially with a concomitant delta-phase reset, in line with entrainment theories [7, 38]. It is likely that working memory mechanisms contribute to the attentional control that is required to benefit from the instruction. While it is interesting to speculate about the mechanisms underlying the benefits in target detection created by an instruction, the data reported here cannot distinguish among the contributions of various attentional and other mechanisms that may underlie the observed instruction effect.

In contrast, reaction times in the present study were not affected by the presence or absence of instruction about the temporal position of the target, and hence appeared unaffected by attention. In both instruction conditions, reaction times were fastest for the temporal cue condition followed by the periodic and aperiodic conditions, like previous research [39]. Although participants were instructed to be fast and accurate, the difficulty of the perceptual task may have led participants to prioritize accuracy, so that explicit information about temporal target position in the condition with instruction may have provided benefits exclusively for accuracy and not for processing time.

A number of studies have behaviorally compared the benefits of single cues, periodic and aperiodic rhythms for the accuracy of auditory target perception. Several studies have assessed the benefits of periodicity for the sensitivity of target perception by comparing performance for a periodic rhythm relative to an aperiodic rhythm [11, 15, 40–44]. These studies showed facilitated target perception for the periodic relative to the aperiodic conditions, in line with our data.

A number of other studies [18, 20, 45; in control patients 19, 46] have compared the perceptual benefits of temporal interval cues with periodic rhythm conditions for targets placed after the termination of the periodic rhythm. Overall, these studies have shown only limited evidence that periodicity may lead to a better behavioral performance than interval cueing. Among those five studies, only one study [20] reported a benefit in reaction times following the rhythmic cue compared to a temporal interval, whereas the other studies did not report any benefit of rhythms over temporal interval cues, neither in sensitivity nor in reaction times. Although the five above-cited studies in one aspect of their design resemble our no-instruction condition (in the sense that no explicit instructions were given in these studies as to when to expect the target), their strategy to present the target stimuli after the termination of a periodic rhythm limits the extent to which their results can be compared with ours. Therefore, it is not certain that applying an instruction to the design of the above-referred studies to direct attention towards the temporal location of the target, would have increased the benefits of periodic over single temporal cue conditions for target perception. Instead, we speculate that the difficulty of the target detection task in our experiment, partially due to inserting the target within the rhythm (as done in [7, 28, 38, 39], may have created in the present study the conditions in which an explicit instruction indicating the temporal location where targets occurred in the periodic rhythm led to sensitivity advantages over single temporal cueing.

To conclude, our results shed light on the frequently proposed idea that target stimuli embedded in a periodic rhythm benefit from automatically generated predictions providing enhanced target processing. We evaluated this possibility by comparing target processing efficiency in periodic rhythms to that afforded by temporal cues and aperiodic rhythms. We exploited the presence or absence of an instruction about the precise temporal location of the target to evaluate whether the advantage afforded by periodicity is necessarily automatic. The main result in our experimental design is that we found no evidence for automatic benefits of periodic rhythms over single temporal cues for target processing. Instead, better target processing in periodic rhythms than after temporal cues only emerged after an explicit task instruction pointing to the precise moment in the rhythm when a target was to be expected. Hence, here, the benefits of periodic rhythms for perception only occurred in interaction with attention. We suggest that our data increase insight into the conditions in which periodic rhythms can provide perceptual advantages to targets embedded in these rhythms. The hypothesized explanatory mechanisms, in which we suggest interactions between oscillatory entrainment and top-down attentional effects should be tested by neurophysiological or electrophysiological experiments [47].

## Supporting information

S1 Table Wilkinson notation of final models. (DOCX)

S1 Fig The size of the temporal shift per participant across six staircase blocks. Decrease of TS size over course of experiment separately per condition, **A**. without receiving an instruction and **B**. instructed when to expect a target in the rhythm. Each staircase preceded a trial block, ensuring sufficient task difficulty in the latter. Group average of to be detected TS decreases over the course of the experiment. During the staircase the TS varied on a fixed step-size of 10 logarithmically spaced steps between 7ms and 1.5 ms. The termination criterion was after 200 trials or 15 reversals. Errorbars depict SEM centered on group mean.
(TIFF)

S2 Fig Instruction when to expect a target improves sensitivity in periodic rhythm compared to a temporal cue. **A**. In the no-instruction condition, participants received no instruction on possible target positions within the temporal sequences and remained unaware that targets occurred always at the same position. In this case sensitivity did not differ between a periodic rhythm and a temporal cue. **B**. When participants were instructed on the temporal contingency of target occurrence in periodic and aperiodic stimuli (participants were instructed in which position to expect the possible presentation of a target), a higher sensitivity for the periodic rhythm compared to temporal cue (as well as aperiodic) occurs. Filled circles depict d’ per participant. Black horizontal line shows the group mean. Errorbars depict bootstrapped confidence intervals (at subject-level). Note the discontinued y-axis for visualization purposes.
(TIF)
