# Supplementary material for: Predictability awareness rather than mere predictability enhances the perceptual benefits for targets in auditory rhythms over targets following temporal cues
Source: PLoS One. 2023 Oct 27;18(10):e0284755. doi: 10.1371/journal.pone.0284755 (PMC10610080; doi:10.1371/journal.pone.0284755)
Supplement: S1 Table — (DOCX) [file pone.0284755.s001.docx]

**Supplementary materials**

**Table T1. Wilkinson notation of final models**

| Large Model  (both experiments)  Sensitivity | d’ ~ Criterion + Target*Temporal Structure*InstructionCondition + (1 \|Subject) -1 |
| --- | --- |
| Large Model  (both experiments)  Reaction times | logRT ~ Temporal Structure*InstructionCondition + (1 \|Subject) |
| No Instruction Exp  Sensitivity | d’ ~ Criterion + Target*Temporal Structure + (1 \|Subject) -1 |
| With Instruction Exp  Sensitivity | d’ ~ Criterion + Target*Temporal Structure + (1 \|Subject) -1 |

**Note**. Criterion is an additional predictor reflecting the intercept

(normally notated as 1, here re-parameterized to -1 to reduce

correlation between fixed effects (see p 262 [35] )
