# Supplementary figures and images for: Predictability awareness rather than mere predictability enhances the perceptual benefits for targets in auditory rhythms over targets following temporal cues
Source: PLoS One. 2023 Oct 27;18(10):e0284755. doi: 10.1371/journal.pone.0284755 (PMC10610080; doi:10.1371/journal.pone.0284755)

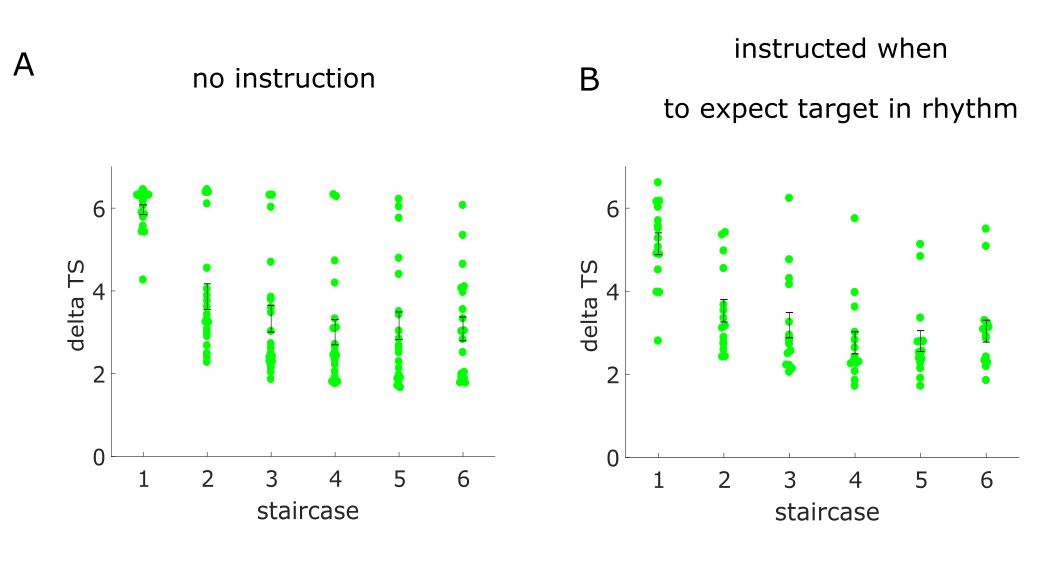

Supplement: S1 Fig — Decrease of TS size over course of experiment separately per condition, A. without receiving an instruction and B. instructed when to expect a target in the rhythm. Each staircase preceded a trial block, ensuring sufficient task difficulty in the latter. Group average of to be detected TS decreases over the course of the experiment. During the staircase the TS varied on a fixed step-size of 10 logarithmically spaced steps between 7ms and 1.5 ms. The termination criterion was after 200 trials or 15 reversals. Errorbars depict SEM centered on group mean. (TIFF) [file pone.0284755.s002.tiff]

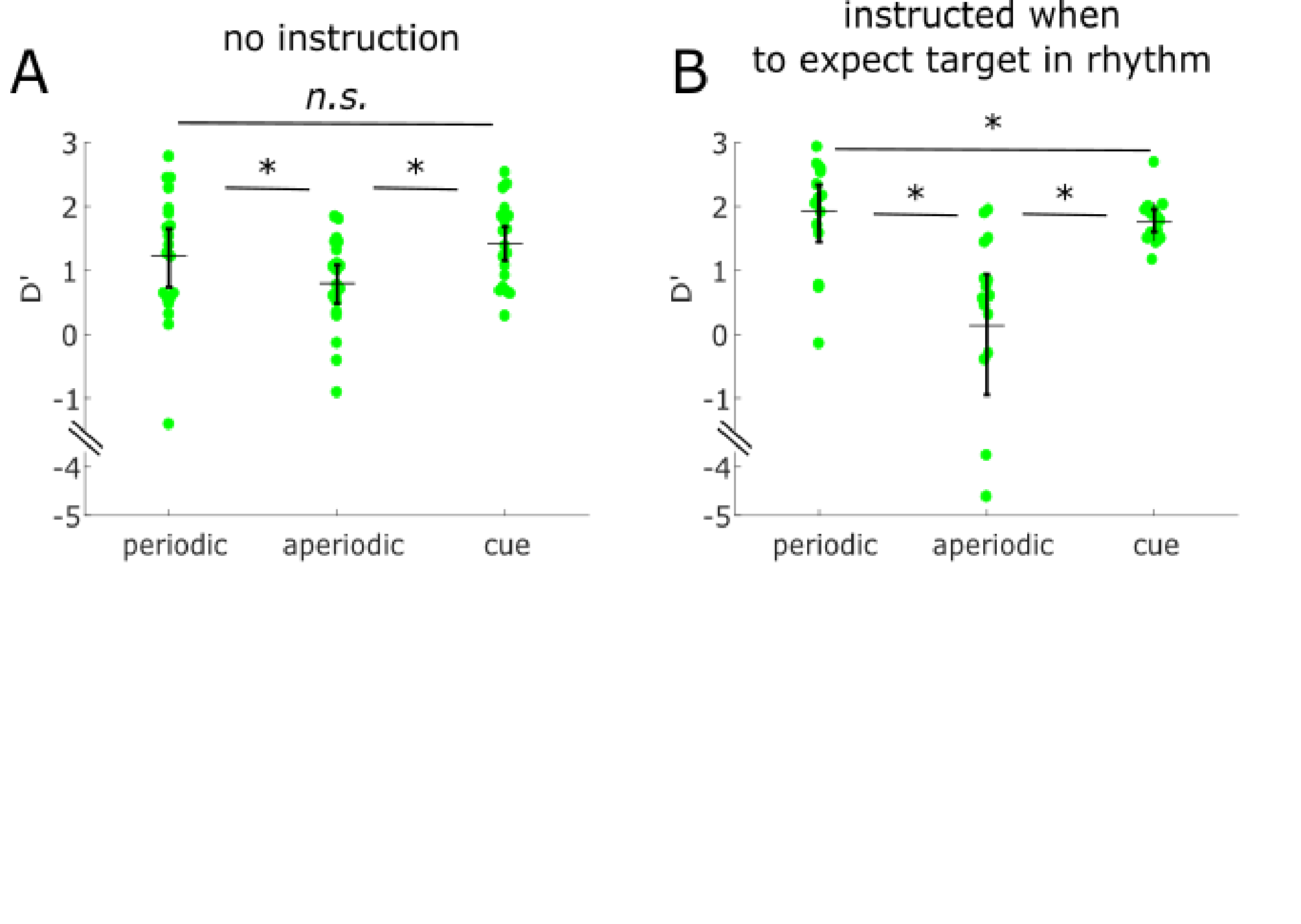

Supplement: S2 Fig — A. In the no-instruction condition, participants received no instruction on possible target positions within the temporal sequences and remained unaware that targets occurred always at the same position. In this case sensitivity did not differ between a periodic rhythm and a temporal cue. B. When participants were instructed on the temporal contingency of target occurrence in periodic and aperiodic stimuli (participants were instructed in which position to expect the possible presentation of a target), a higher sensitivity for the periodic rhythm compared to temporal cue (as well as aperiodic) occurs. Filled circles depict d’ per participant. Black horizontal line shows the group mean. Errorbars depict bootstrapped confidence intervals (at subject-level). Note the discontinued y-axis for visualization purposes. (TIF) [file pone.0284755.s003.tif]
